# Autophagy-Regulated ROS from Xanthine Oxidase Acts as an Early Effector for Triggering Late Mitochondria-Dependent Apoptosis in Cathepsin S-Targeted Tumor Cells

**DOI:** 10.1371/journal.pone.0128045

**Published:** 2015-06-01

**Authors:** Chien-Chang Huang, Cheng-Che Lee, Hsiao-Han Lin, Mei-Chi Chen, Chun-Cheng Lin, Jang-Yang Chang

**Affiliations:** 1 National Institute of Cancer Research, National Health Research Institutes, Tainan, Taiwan, ROC; 2 The Institute of Basic Medical Sciences, College of Medicine, National Cheng Kung University, Tainan, Taiwan, ROC; 3 Department of Chemistry, National Tsing Hua University, Hsinchu, Taiwan, ROC; 4 Division of Hematology and Oncology, Department of Internal Medicine, National Cheng Kung University Hospital, College of Medicine, National Cheng Kung University, Tainan, Taiwan, ROC; 5 Institute of Molecular Medicine, College of Medicine, National Cheng Kung University, Tainan, Taiwan, ROC; Swedish Neuroscience Institute, UNITED STATES

## Abstract

Cathepsin S (CTSS), which is highly expressed in various malignant tumor cells, has been proposed to promote tumor progression, migration, and invasion. CTSS inhibition not only blocks tumor cell invasion and endothelial tube formation but also induces cellular cytotoxicity. In our previous studies, we have observed that CTSS inhibition induces autophagy, which is responsible for up-regulating xanthine oxidase for early ROS generation and consequent cell death. However, whether the autophagy-regulated early ROS triggers apoptosis remains unclear. We conducted a long-term follow-up study to investigate the relationship between early autophagy and late mitochondria-dependent apoptosis. We demonstrated that early ROS generation is critical for mitochondria damage and the activation of intrinsic apoptotic pathway. Attenuating the early ROS level diminished later mitochondrial damage and downstream apoptotic signaling. Collectively, mitochondria-dependent apoptosis is regulated by autophagy-regulated early ROS, which serves as an early effector that triggers mitochondrial signaling for late apoptosis. The data emphasize the essential role of autophagy-regulated early ROS in triggering late apoptotic signaling.

## Introduction

The cysteine proteases, which represent the major group of cathepsins, have recently been indicated to associate with tumor metastasis and recurrence [1–4]. Cathepsin S, also known as CTSS, contains an active cysteine residue in the active site for the turnover of intracellular and extracellular proteins. In addition to being expressed in antigen-presenting cells [5, 6], CTSS has recently been reported to be overexpressed in various malignant tumor cells [7–11]. Thus, CTSS activities have emerged as a potential therapeutic target for cancer treatment. In recent years, a series of small molecule inhibitors have been developed, and they have shown promising effects in inhibiting the spread of malignant cells and in promoting tumor cell death [12–14]. Similarly, targeting CTSS activities by using the specific monoclonal antibody Fsn0503 not only attenuates tumor invasion and HUVEC tube formation but also elicits strong antibody-dependent cellular cytotoxicity in tumor cells [15–17].

Autophagy, an evolutionarily conserved process in eukaryotic cells, is initiated with the formation of the phagophore, which expands and grows to engulf the cytosolic components, and then becomes an autophagosome with an enclosed double-membrane structure [18]. In addition to homotypically fusing with other autophagosomes, the autophagosome can fuse with lysosomes to form an autolysosome for digestion. Molecularly, autophagosomes is induced by class III phosphatidylinositol 3-kinase (PI3K), and is tightly regulated by a group of genes known as autophagy-related genes (ATG). Of these ATGs, the most understood is ATG8, also called LC3 in mammalian cells. After the initiation of autophagy, the cytosolic form of LC3 (LC3-I) is proteolytically cleaved and lipidated to phosphatidylethanolamine (PE), forming LC3-II, which translocates rapidly onto the autophagosomal membrane in a punctate distribution. Thus, lipidated LC3-II is a valuable marker indicating the presence of autophagosomes and autophagy activation. In addition to its essential quality-control function in cells, autophagy can be activated in different environmental stress conditions, enabling cells to degrade macromolecules and organelles [19–21]. The degrading process generates free amino acids and fatty acids that can be recycled to maintain the synthesis of proteins necessary for cell survival. Therefore, autophagy occurs rapidly in starved cells when metabolic demands increase, but cannot be immediately supplemented. In addition, autophagy is responsible for the turnover of aggregated proteins and the removal of damaged organelles such as damaged mitochondria when cells respond to environmental toxins, chemotherapeutic drugs, and aging. Thus, the activation of autophagy is commonly considered a cell survival mechanism.

Although autophagy is typically considered to be a protective mechanism for cell survival, recent studies have reported different observations, stating that autophagy plays a potential cytotoxic role in the cell death process [22–24]. Three major types of cell death exist, as defined based on morphological and molecular criteria [25]. Apoptotic cell death is characterized by basic morphologic changes such as cell shrinkage, decreased nuclear size, chromatin condensation, and DNA fragmentation. The molecular genetic markers for apoptosis include caspase activation, mitochondria-dependent signaling transduction, and the translocation of phosphatidylserine from the cytoplasmic face of the plasma membrane to the cell surface. Necrosis is another form of cell death characterized by the breakdown of the plasma membrane for the spillage of cytoplasmic contents, the swelling of cellular organelles, and the release of inflammatory cellular contents [26]. Distinct from apoptotic and necrotic cell death, however, the term “autophagic cell death” remains controversial. It is contradictory but also plausible that the autophagy-induced excessive degradation of cellular components disrupts the homeostatic balance between biosynthesis and degradation. Thus, autophagic cell death is often characterized by the sustained activation of autophagy in dying cells [27–29].

We recently demonstrated that inhibiting CTSS activities in tumor cells can rapidly induce autophagy [30] and act as an upstream event for mediating early ROS production through xanthine oxidase (XO) [31]. Although this autophagy-regulated ROS may suffice for DNA damage, resulting in cell death, whether CTSS inhibition can trigger cell death via the apoptotic pathway remains unknown. For this study, we conducted an in-depth examination of the sequential order and interrelationships between XO-dependent early ROS generation and mitochondrial damage regarding mitochondria-dependent late ROS production (second oxidative burst), caspase-9/-3 signaling cascades, and the intrinsic mitochondrial apoptotic pathway in cells treated with CTSS inhibitors.

## Materials and Methods

### Cell cultures and Chemicals

The OEC-M1 cells, derived from a human oral epidermoid carcinoma, were established and provided by Dr. Ching-Liang Meng [32, 33]. OEC-M1 cells were maintained in RPMI1640 (Gibco) supplemented with 10% fetal bovine serum, 100 U/mL of penicillin, and 100 μg/mL of streptomycin at 37°C in an atmosphere comprising 95% air and 5% CO_2_. The CTSS inhibitor 6r was synthesized and provided by Professor Chun-Cheng Lin at the Department of Chemistry, National Tsing Hua University, Hsinchu, Taiwan. The Z-FL-COCHO (ZFL) was purchased from Calbiochem (#219393). The 3-methyladenine (3-MA, #M9281), 2',7'-dichlorofluorescin diacetate (DCFH-DA, #D6883), allopurinol (#A8003), CCCP (#C2759), chloroquine (CQ, #C6628), and bafilomycin A1 (BAF, #B1793) were purchased from Sigma. The JC-1 (#T3168) and MitoSOX Red (#M36008) were purchased from Molecular Probes. CytoPainter MitoGreen (#ab176830) was obtained from abcam.

### Antibodies

Antibodies against Bax (#5023), Caspase-3 (#9662), Caspase-9 (#9502), COX-IV (#4844), SQSTM1/p62 (#5114), and LC3B (#3868) were purchased from Cell Signaling Technology. Antibodies against ACTIN (#MAB1501) and cytochrome c (#556433) were obtained from Millipore and BD Pharmingen, respectively. The primary antibody against LC3 (#L7543) was obtained from Sigma. Anti-xanthine oxidase (#sc-20991) antibodies were acquired from Santa Cruz.

### Western blot analysis

In brief, analyzed cells were collected by conducting centrifugation at 800 x *g* for 5 min at 4°C, washed twice with ice-cold PBS, and resuspended in a lysis buffer (Sigma, #C2978) containing 0.5 mM PMSF, 1 mM Na_3_VO_4_, and 1X protease inhibitor cocktail (Roche, #04693116001). After 10 min of incubation at 4°C, the samples were sonicated and centrifuged at 14 000 rpm for 10 min. The protein concentration was analyzed using the BCA Protein Assay Kit (Pierce, #23225). An equal amount of proteins were subjected to standard sodium dodecyl sulfate polyacrylamide gel electrophoresis (SDS-PAGE), and were then transferred onto PVDF membranes. After blocking with tris-buffered saline (TBS) containing 5% nonfat milk powder, the membranes were probed with primary antibodies, as described individually in the figure legends. The blotted membranes were subsequently washed twice with TBST (TBS containing 0.1% of Tween 20) for 10 min. The membrane-bound primary antibodies were visualized using secondary antibodies conjugated with horseradish peroxidase (Santa Cruz). The chemiluminescent signal was visualized using Western Lightning Plus ECL (Perkin Elmer) with an appropriate time of exposure to X-ray films.

### Flow cytometric analysis of apoptosis by Annexin V-propidium iodide double staining

Apoptotic cells were stained and analyzed using the FITC Annexin V Apoptosis Detection Kit (BD Pharmingen, #556547), according to the manufacturer’s instructions. In brief, the cells were treated with inhibitors for a set time, washed twice with ice-cold PBS, and subsequently resuspended in a 1X Binding Buffer. The 1 x 10^5^ cells were transferred into a new tube and stained with FITC Annexin V and propidium iodide (PI) at 25°C in the dark. After 15 min of incubation, 400 μL of the 1X Binding Buffer was added into each tube, which was subsequently analyzed by flow cytometry. For each assay, 10 000 cells were collected and analyzed using BD FACSCalibur with Cell Quest software for acquisition and analysis. The results obtained in this study are presented as the mean ± SD in triplicate.

### TUNEL assays for apoptosis detection

TUNEL analysis was performed according to the manufacturer’s instructions (Roche, #11684795910). In brief, the cultured cells were cultivated on CultureSlides (BD Biosciences) and treated with inhibitors for a set time. The cells were subsequently washed 3 times with ice-cold PBS and fixed in a freshly prepared fixation solution (4% paraformaldehyde). After 10 min of fixation, the cells were washed twice with PBS and permeabilized in a freshly prepared permeabilization solution (0.1% Triton X-100, 0.1% sodium citrate) for 10 min. Afterward, the cells were incubated with a TUNEL reaction mixture for 60 min at 37°C in a humidified atmosphere in the dark. After washing twice with PBS, the samples were mounted using a ProLong antifade reagent (Molecular Probes, #36934) and were imaged using a Leica DMI 4000B microscope.

### Assessment of autophagy activation by immunodetection

Cells were grown on CultureSlides (BD Biosciences) and exposed (or not) to 20 μM of 6r or ZFL for 24 h. To detect autophagic vacuoles, the cells were subsequently incubated with 0.05 mM of monodansylcadaverine (MDC) in PBS at 37°C for 10 min. Afterward, the cells were washed with ice-cold PBS, and the fluorescence intensities were obtained immediately using a Leica DMI 4000B microscope. For the immunodetection of LC3 puncta, the specimens were fixed with 4% paraformaldehyde, washed with ice-cold PBS, permeabilized with Triton X-100, blocked with 1% BSA, and subsequently incubated with the LC3 primary antibody (Cell signaling, #3868) for an additional 16 h. Bound antibodies were visualized by incubating the specimens with anti-rabbit secondary antibodies (Molecular Probe, #21207) for 2 h. To visualize the nuclei, DAPI was employed as a counterstain.

### Flow cytometric analysis of the mitochondrial membrane potential

The mitochondrial membrane potential was determined using JC-1, which accumulates as aggregates inside the mitochondria with red fluorescence, but is localized outside the mitochondria in a monomeric state with green fluorescence. For positive control, cells were incubated with CCCP for 10 min to disrupt the membrane potential. After drug treatment, the cells were trypsinized, washed twice with ice-cold PBS, and suspended in 1 mL of a preheated medium containing JC-1 (5 μg/mL) for 30 min at 37°C in the dark. The red and green fluorescence intensity was quantified using a FACSVantage flow cytometric analyzer (Becton Dickinson FACSCalibur). For each assay, 10 000 events were collected and analyzed. The results obtained in this study are presented as the mean ± SD in triplicate.

### Analysis of free radical production

Intracellular ROS production was measured as previously described [31]. The ROS elevation was determined using DCFH-DA or MitoSOX, and the fluorescent intensity was quantified by using flow cytometry. In brief, the cells were seeded onto 6-cm culture plates and allowed to grow overnight to reach an approximate confluence. After treatment with cathepsin S inhibitors or with other pharmacological inhibitors for the indicated times, the cells were further incubated with 25 μM of DCFH-DA or 5 μM of MitoSOX at 37°C in a CO_2_ incubator for 30 min. The cultured cells were washed, trypsinized, neutralized, and resuspended in appropriate amounts of PBS. The fluorescence intensity was quantified using a FACSVantage flow cytometric analyzer (BD FACSCalibur). For each analysis, 10 000 events were analyzed. The results obtained in this study are presented as the mean ± SD in triplicate. To monitor mitochondrial ROS production, CytoPainter MitoGreen and MitoSOX were used to label mitochondria and mitochondria-specific ROS, respectively. After 24 h of 6r treatment, the cells were further incubated with CytoPainter MitoGreen (at 1:1000 ratio) and 5 μM of MitoSOX red at 37°C in a CO_2_ incubator for 30 min. Cells were washed twice with PBS and live cell imaging was immediately performed by Leica DMI 4000B microscope with appropriate, filters and lasers.

### Preparation of mitochondrial and cytosolic fractions

Mitochondrial and cytosolic fractions were prepared according to the manufacturer’s instructions (G-Biosciences, #786–022). In brief, the cells were treated with or without inhibitors for a set time, washed twice with ice-cold PBS, and subsequently resuspended in 500 μL of ice-cold SubCell Buffer-I. After homogenizing the cells by using the Dounce homogenizer, the lysates were transferred into a microcentrifuge tube. The Dounce homogenizer was washed with an additional 200 μL of ice-cold SubCell Buffer-I and pooled together. To obtain a 1X final concentration of SubCell Buffer-II, 350 μL of 3X SubCell Buffer‐II was added to the solution, mixed well, and then centrifuged in a tube at 800 x *g* for 10 min to pellet the nuclei. The supernatant was transferred to a new tube and centrifuged at 12 000 x *g* for 20 min to pellet the mitochondria. The supernatant (cytosolic fraction) was transferred to a new tube and stored at -80°C. The pellet containing the mitochondria was washed with an additional 500 μL of 1X SubCell Buffer‐II and then resuspended in a 1X lysis buffer. The protein concentrations of the mitochondrial and cytosolic fractions were further analyzed using the BCA Protein Assay Kit (Pierce, #23225).

### Silencing xanthine oxidase genes by specific siRNA

The XO-specific siRNAs (sc-41691) and non-targeting siRNA (scramble control, sc-37007) were all purchased from Santa Cruz. The silencing approach was performed by using Lipofecamine RNAiMAX reagent (Invitrogen) in Opti-MEM, according to the manufacturer’s instructions. Briefly, cells grown on 6-well culture plates were transfected with a well-optimized siRNA mixture, which contained a siRNA duplex and 7.5 μL of transfection reagent, yielding 200 μL of the transfection medium (final siRNA concentration of 25 nM). After 5 h of transfection, the media were aspirated and replaced with 2 mL of fresh RPMI containing 10% FBS for further culturing for 24 h.

### Nuclear size counting

After cells were treated with 6r for indicated time points, the cells were fixed with 4% paraformaldehyde, stained with DAPI, and subsequently imaged using a Leica DMI 4000B microscope. The counting of DAPI-stained nuclei was performed colorimetrically and the individual size of nuclei was further calculated by using the software Image-Pro. Nuclear area was defined as the area within the outlined nuclear perimeter.

### Statistical analyses

Statistical analysis was performed using Student’s *t* test. The results are expressed as the means ± SD. In this study, the *P* values are represented by asterisks *P < 0.05, **P < 0.01, and ***P < 0.001. A *P* value < 0.05 was considered statistically significant.

## Results

### Inhibition of CTSS activities by 6r and ZFL induce apoptotic and autophagic hallmarks

**Fig 1:**
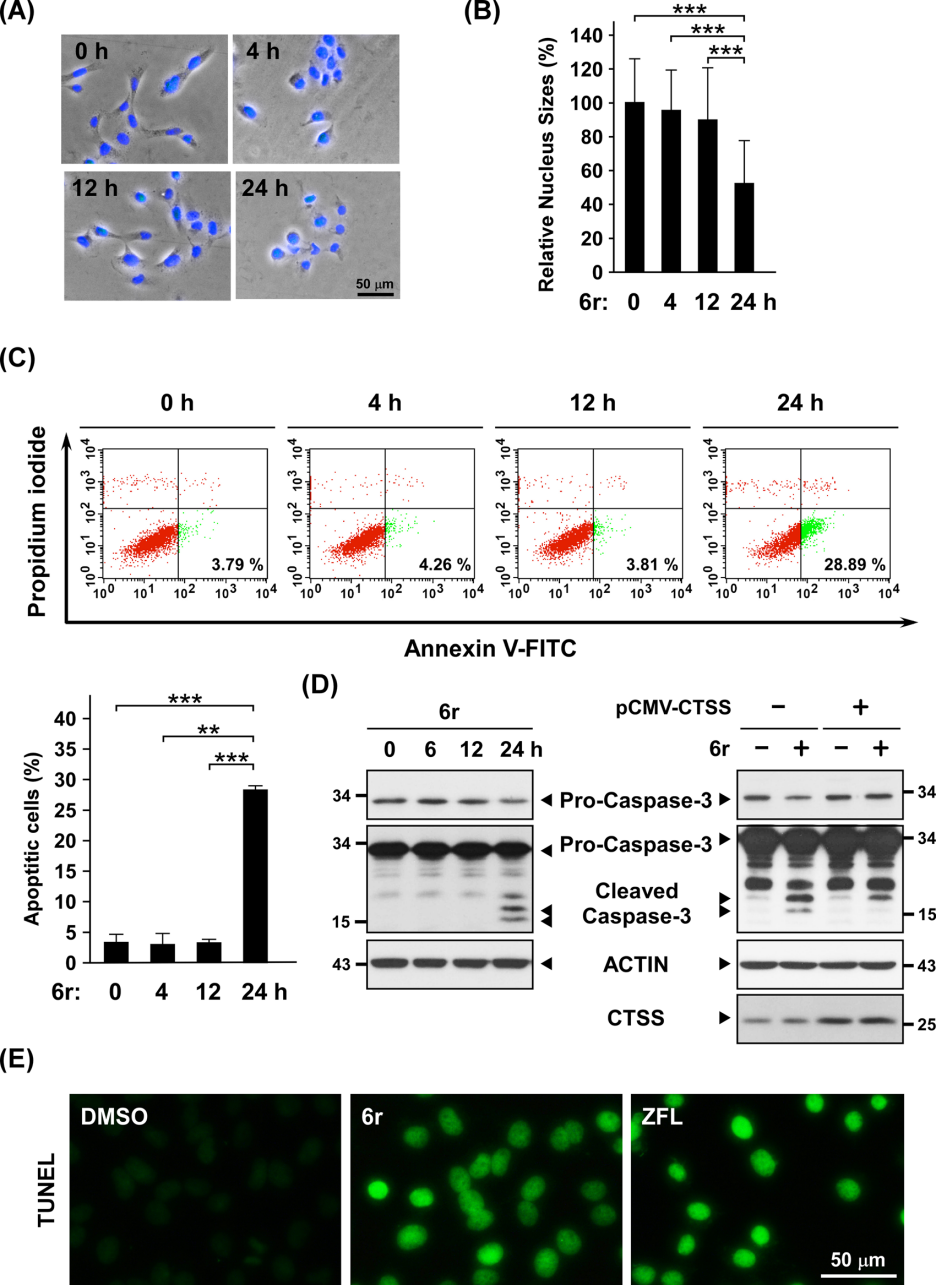
Inhibition of CTSS activities induces apoptotic hallmarks. (A) After OEC-M1 cells were incubated with DMSO or 20 μM of the CTSS inhibitor 6r for the indicated time points, the cells were fixed with 4% paraformaldehyde, and stained with DAPI. The cell morphology and nucleus were monitored under a phase-contrast and a fluorescent microscope, respectively. Merged images are shown. (B) Cells were treated with 20 μM of 6r at different times. Nuclei were stained with DAPI, and images were taken using the fluorescent microscope. Relative nuclear sizes were analyzed using the Image Pro Plus software. Nuclear area was defined as the area within the outlined nuclear perimeter. Differences were found to be statistically significant at ***P < 0.001. (C) Time-course analysis of apoptosis during 6r treatment. Cells were exposed to 20 μM of 6r at the indicated time points, harvested, and stained with Annexin V and PI, as described in the materials and methods section. The numbers of apoptotic cells were quantified using flow cytometry. Data shown in the below panel are representative of 3 independent experiments. Differences were found to be statistically significant at **P < 0.01 and ***P < 0.001. (D) Left panel: Cells were treated with 20 μM of 6r for various durations, and cleaved caspase-3 fragments were analyzed through western blotting. The upper panel indicates short-term exposure of the blot. ACTIN was shown as an internal control for semiquantitative loading in each lane. Right panel: After OEC-M1 cells were transfected with pCMV vector or pCMV-CTSS for 24 h, cells were subsequently treated with 20 μM of 6r for additional 24 h. Compared to vector control cells, cells with increased CTSS expression showed reduced caspase-3 activation upon 6r treatment. (E) Representative images of a TUNEL assay in cells treated with 20 μM of 6r or ZFL for 24 h. Apoptotic cells display TUNEL-positive nuclei stained green.

We first examined whether the pharmacological inhibition of CTSS by 6r induces cell cytotoxicity accompanied by apoptosis and autophagy. As shown in Fig 1A, cell shrinkage was observed in 24 h of treatment with 6r. The morphology of the cells and the average size of the nucleus before the 12-h treatment with 6r remained unchanged. However, a significant reduction in nuclear size was observed after 24 h of treatment with 6r (Fig 1B, *P* = 4 x 10^–5^ compared with 12 h of treatment). To validate the morphological changes associated with apoptosis, we further examined apoptotic cell death by conducting Annexin V/PI double staining. In this analysis, cells with single-positive Annexin V staining were considered early apoptotic cells, whereas cells with single-positive PI staining were considered necrotic cells. As shown in Fig 1C, no increase in Annexin V single-positive cells occurred before 12 h of treatment with 6r. However, the number of apoptotic cells (Annexin V single-positive) increased significantly after 24 h of treatment with 6r (*P* = 2 x 10^–6^). The apoptotic signaling pathway ends at the point of the execution phase with the activation of executive caspase-3 [34]. In parallel, therefore, apoptotic hallmarks were examined by determining the amount of cleaved caspase-3 in cells. As shown in Fig 1D (left panel), no cleaved form of caspase-3 was observed in cells before 12 h of treatment with 6r. A substantially cleaved form of caspase-3 was observed in cells after exposure to 6r for 24 h. To further determine whether 6r-induced apoptosis was a consequence of CTSS inhibition, a complementing rescue experiment was performed. Cells with or without CTSS overexpression were treated with 6r for 24 h and then subjected to Western blotting analysis for caspase-3 activation. As shown in the right panel of Fig 1D, increased amount of cleaved caspase-3 was observed in vector-transfected cells after 24 h treatment of 6r. In contrast, the level of cleaved caspase-3 reduced in CTSS-overexpression cells after 24 h of treatment with 6r, indicating the observed effects of 6r are indeed results of CTSS inhibition. Additionally, CTSS inhibition-induced apoptotic cell death was corroborated by TUNEL assays. As shown in Fig 1E, increased TUNEL intensities were observed after exposure to 6r for 24 h. Moreover, treating cells with another CTSS inhibitor, ZFL, also showed increased TUNEL intensities. These results suggested that the prolonged pharmacologic inhibition of CTSS by 6r can induce apoptosis.

**Fig 2:**
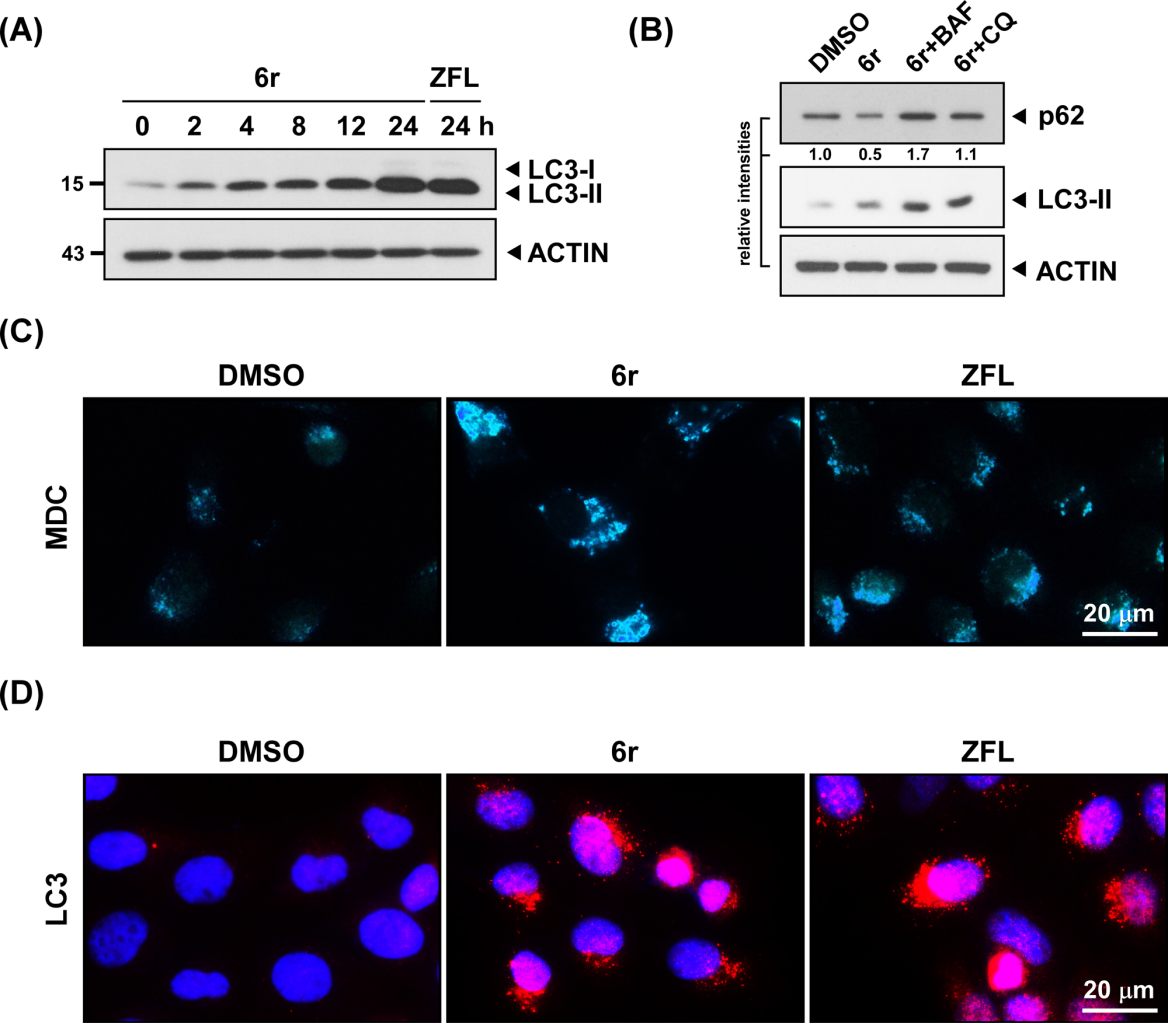
Autophagy is uninterruptedly up-regulated in CTSS-inhibiting cells. (A) The OEC-M1 cells were treated with 20 μM of 6r or ZFL for the indicated times. Autophagy activation was evaluated with the detection of Lipidated LC3-II accumulation. The amount of loading proteins was assessed with anti-ACTIN antibodies. (B) Autophagy inhibition by CQ or BAF suppressed 6r-induced p62 degradation. The relative band intensities are shown. (C) The inhibition of CTSS by 6r or ZFL induced the autophagic vacuolization of MDC-labeled vesicles. After treatment with 20 μM of 6r or ZFL for 24 h, the cells were further incubated with MDC for 10 min, and immediately analyzed using fluorescent microscopy. (D) The visualization of autophagosomes by LC3 puncta formation in CTSS-inhibiting cells. Cells were treated with 20 μM of 6r or ZFL for 24 h, and then processed for immunofluorescence by using an antibody against the LC3 protein. Aggregated LC3 proteins appeared as puncta structures in cells.

We then examined whether autophagy continued to exist in these apoptotic cells after prolonged 6r treatment. Autophagic hallmarks were first determined using western blotting for LC3-II accumulation. As shown in Fig 2A, the amounts of LC3-II increased in as early as 2 h and incessantly in cells even after 24 h of exposure to 6r. Similarly, treating cells with another CTSS inhibitor, ZFL, for 24 h caused an incremental increase of LC3-II. To further confirm autophagic flux in 6r-targeted OEC-M1 cells, monitoring autophagic degradation of p62/SQSTM1 was performed. As shown in Fig 2B, decreased p62 level and increased LC3-II amount were observed in 6r-treated cells. Furthermore, co-treatment with chloroquine (CQ) or baflilomycin A1 (BAF) led to an increase in both p62 and LC3-II level, supporting p62 degradation and LC3-II increment primarily through the autophagic flux. In addition, autophagosomal vacuoles, another autophagy marker, were determined using the autofluorescent drug MDC [35]. As shown in Fig 2C, small and few MDC-labeled vacuoles were observed in DMSO-treated control cells, indicating basal autophagy. However, increased sizes and numbers of autophagosomal vesicles were observed after 24 h of CTSS inhibition by either 6r or ZFL, indicating that prolonged CTSS inhibition caused a large accumulation of autophagosomal vesicles in cells. Furthermore, 6r- or ZFL-induced persistent autophagy was corroborated by directly immunostaining the formation of LC3 puncta structures. As shown in Fig 2D, the numbers of LC3 immunopositive dot-like structures (red dots) increased significantly in either 6r- or ZFL-treated cells. Overall, these results show that, after prolonged CTSS inhibition by either 6r or ZFL, the cells concurrently displayed apoptotic and autophagic hallmarks.

### CTSS inhibition by 6r induces the second oxidative burst from mitochondria

**Fig 3:**
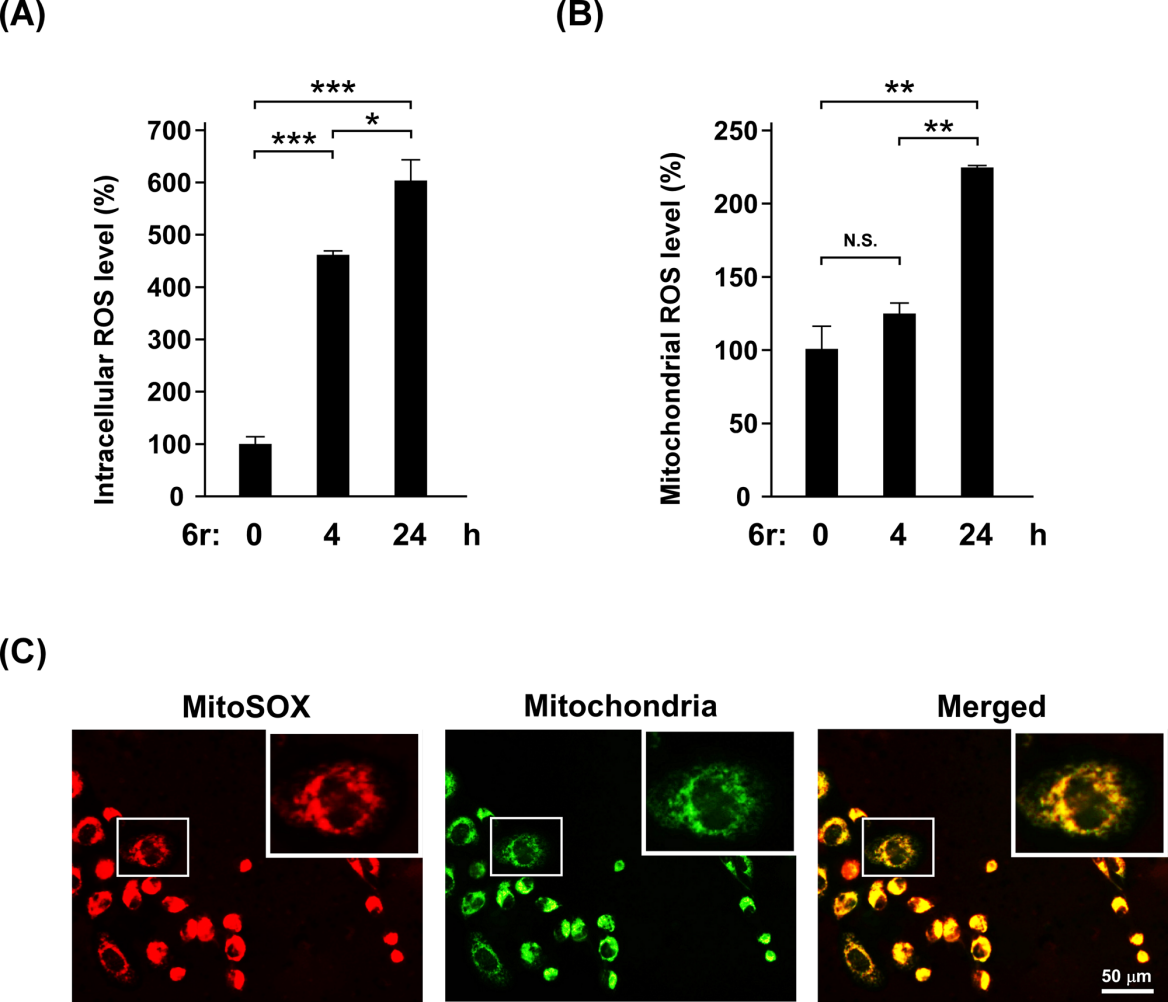
Prolonged inhibition of CTSS by 6r causes a second oxidative burst from the mitochondria. (A) Cells were incubated with 20 μM of 6r for the indicated times, and intracellular ROS levels were detected using DCFH-DA, and were quantified through flow cytometry. The data represent the mean ± SD of 3 independent experiments. (B) After treatment with 20 μM of 6r for the indicated times, elevated mitochondria-generated ROS were identified using MitoSOX, and were quantified using flow cytometry. The data represent the mean ± SD of 3 independent experiments. Differences were found to be statistically significant at *P < 0.05, **P < 0.01, and ***P < 0.001. N.S. denotes no significant difference. (C) Cells were treated with 20 μM of 6r for 24 h, and then labelled with CytoPainter MitoGreen and MitoSOX red. Yellow color in the merged figure denotes mitochondria that are actively producing ROS.

In our previous study [31], early ROS rapidly generated from autophagy-regulated XO but not from the mitochondria in 6r-treated cells. To investigate whether prolonged early ROS generation is sufficiently harmful to cause mitochondrial damage and subsequently induce the second oxidative burst from the mitochondria, we dissected the time-course ROS generation by using a general ROS indicator, DCFH-DA, and a mitochondria-specific ROS indicator, MitoSOX Red. Intracellular ROS generation was first measured using DCFH-DA, which was cleaved by intracellular esterases to form DCFH, and then oxidized by cellular radicals to form fluorescent DCF. Consistent with our previous report, 6r rapidly induced early ROS production within 4 h (Fig 3A). Remarkably, a second oxidative burst (an approximate 90% increment) was observed in cells after 24 h of treatment with 6r compared with 4 h of treatment with 6r. To further examine whether the second oxidative burst originated in the mitochondria, we used MitoSOX Red, a probe of mitochondrial superoxide production in living cells, to determine mitochondria-derived ROS generation. As shown in Fig 3B, compared with the control cells, the relative fluorescent signal of MitoSOX Red remained unaltered (*P* = 0.11) in cells after 4 h of treatment with 6r. However, an approximate 104% increment of MitoSOX red fluorescence was detected in cells after 24 h of treatment with 6r compared with cells that underwent 4 h of treatment with 6r. Overall, the results suggested that 6r quickly induced early ROS generation from XO (≤4 h), whereas prolonged 6r treatment (≥24 h) triggered the second oxidative burst from the mitochondria. Next, we determined the localization of MitoSOX fluorescence in cells. As shown in Fig 3C, the cytosolic location of the MitoSOX signal was observed. In addition, MitoSOX Red fluorescence was colocalized with mitochondria, which labeled by CytoPainter MitoGreen.

### CTSS inhibition by 6r induces mitochondrial damage

**Fig 4:**
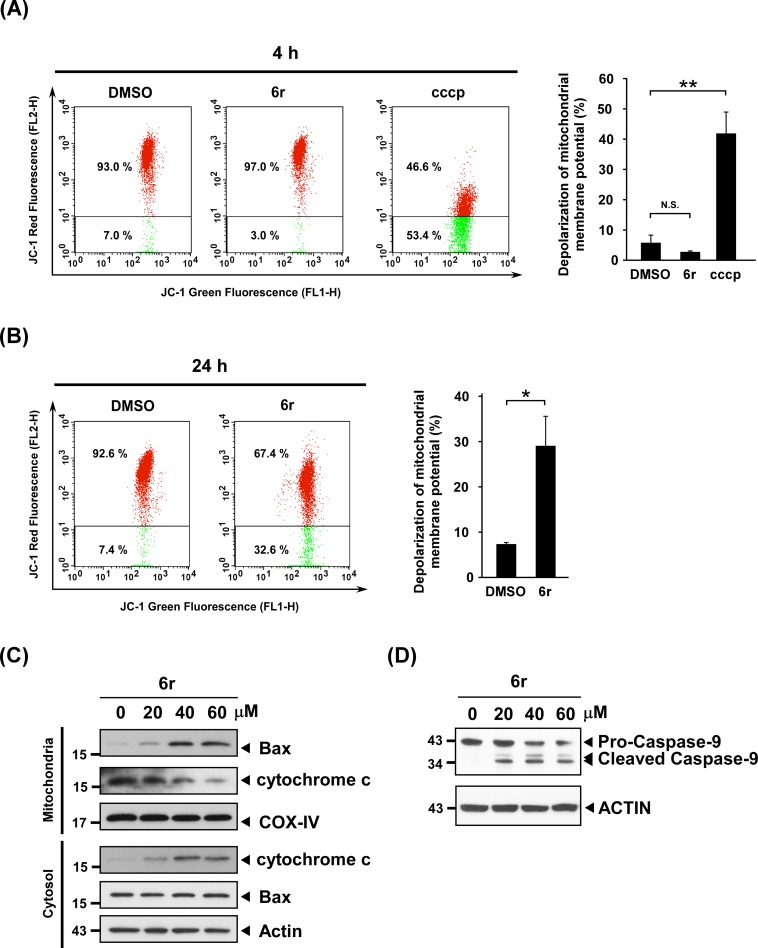
CTSS inhibition by 6r causes mitochondrial damage. (A) The mitochondrial membrane potential was determined using JC-1, and was quantified using flow cytometry. Cells were treated with 20 μM of 6r for 4 h, and then stained with JC-1 for 30 min. The JC-1 aggregated form exhibited red fluorescence, indicating high membrane potential, whereas the JC-1 monomer form exhibited green fluorescence, indicating the collapse of membrane potential. CCCP was used as a positive control. Differences were found to be statistically significant at **P < 0.01. N.S. denotes no significant difference. (B) The inhibition of CTSS by 6r for 24 h induced the collapse of the mitochondrial membrane potential. Differences were found to be statistically significant at *P < 0.05. (C) Cells were exposed to 6r at the indicated concentrations for 24 h. Mitochondrial and cytosolic fractions were prepared as described in the materials and methods section, and were subjected to western blot analysis for cytochrome c, Bax, ACTIN, and COX-IV. ACTIN and COX-4 were used as the internal control for cytosolic and mitochondrial fractions, respectively. (D) Cells were treated with 6r for the indicated concentrations, and cleaved caspase-9 fragments were analyzed with western blotting. ACTIN was shown as an internal control for semiquantitative loading in each lane.

During mitochondria-dependent apoptosis, mitochondrial damage accompanies mitochondrial depolarization and the release of apoptotic factors for triggering the activation of the downstream apoptotic signaling pathway. The cationic dye 5,5',6,6'-tetrachloro-1,1',3,3'-tetraethylbenzimidazolylcarbocyanine iodide (JC-1) is widely used to detect the mitochondrial membrane potential in the early stage of apoptosis [36, 37]. Therefore, we examined changes to the mitochondrial membrane potential in 6r-treated cells by using JC-1 staining. In living cells, JC-1 localizes to the cytoplasm in green fluorescent monomeric form. In the presence of polarized mitochondria, JC-1 exhibits potential-dependent accumulation in the mitochondria as polymeric forms (JC-1 aggregation) and emits red fluorescence. Therefore, decreased mitochondrial membrane potential results in the decreased aggregation of JC-1 in the mitochondria, accompanied by decreased red fluorescent intensities. In this study, the mitochondrial uncoupler carbonyl cyanide 3-chlorophenylhydrazone (CCCP) was used as a positive control. As shown in Fig 4A, the addition of CCCP effectively dissipates the mitochondrial membrane potential. However, the mitochondrial membrane potential remained unaltered in cells treated with 6r for less than 4 h. Furthermore, the pattern of the red fluorescence of JC-1 apparently shifted in cells after 24 h of treatment with 6r compared with the DMSO-treated control cells (Fig 4B), indicating the loss of the mitochondrial membrane potential. A dysfunction of the mitochondrial membrane not only causes increments in mitochondrial membrane permeabilization and ROS leakage but also facilitates the release of cytochrome c [38]. In addition, the Bax protein was reported to form a proteolipid pore in the mitochondrial outer membrane to allow cytochrome c release from mitochondria [39, 40]. Thus, we examined whether 6r treatment triggers Bax translocation into the mitochondria and the release of cytochrome c to the cytosol. Our data showed that increment of Bax was observed in the mitochondrial fractions after 24 h of exposure with 6r. Moreover, the level of cytosolic cytochrome c increased in a concentration-dependent manner after 24 h of treatment with 6r (Fig 4C). During mitochondria-dependent apoptosis, the activation of caspase-9 subsequently occurred, thereby triggering a caspase signaling cascade. Thus, caspase-9 activation was assessed by referencing an increase in the level of its cleaved forms. As shown in Fig 4D, 6r treatment raised the levels of cleaved caspase-9. Overall, these observations indicate that CTSS inhibition induced by 6r causes mitochondrial damage and activates the mitochondria-dependent apoptotic signaling pathway.

### Early ROS is responsible for subsequent mitochondrial damage and the triggering of the mitochondrial apoptotic signaling pathway

**Fig 5:**
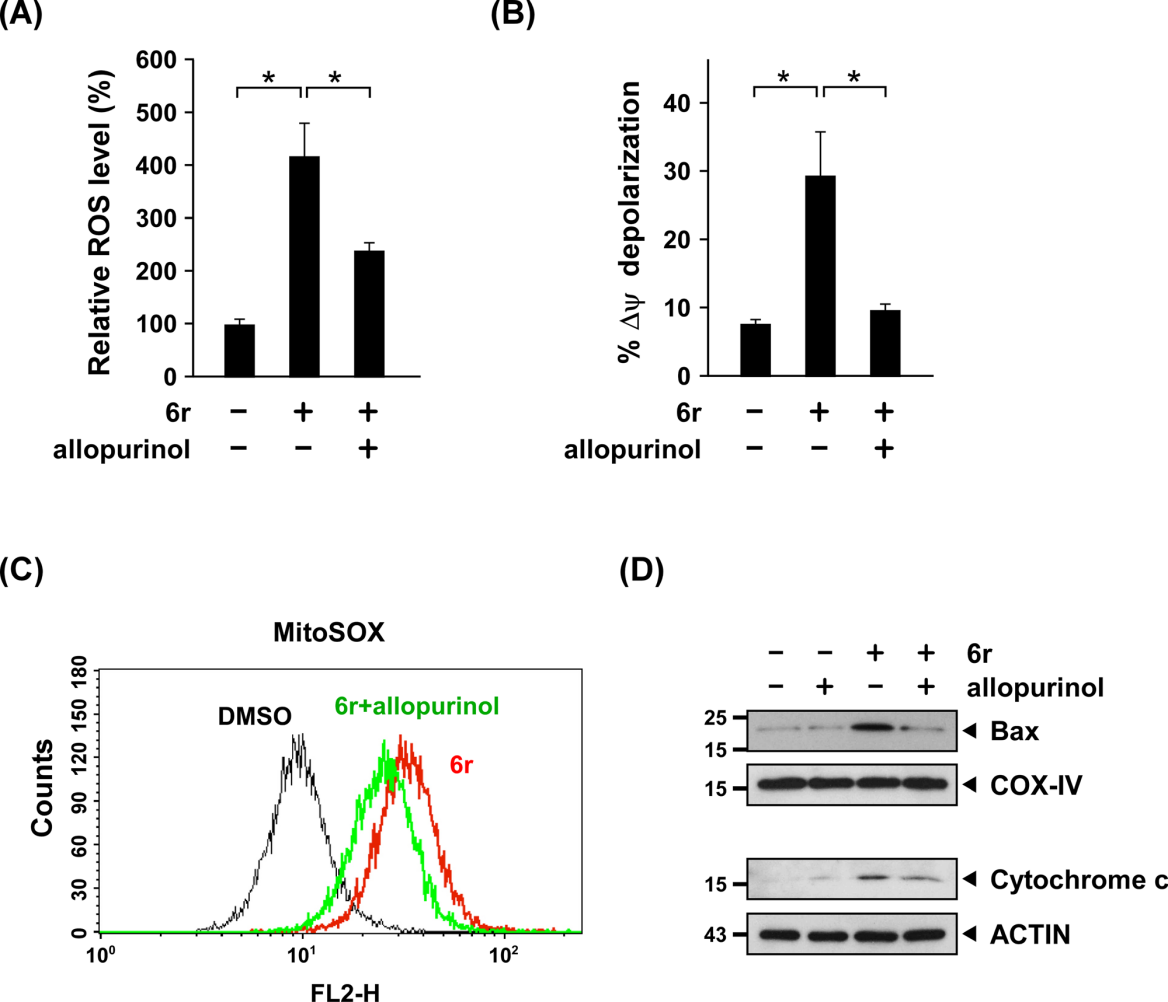
CTSS inhibition-induced early ROS is responsible for mitochondrial damage and the second oxidative burst. (A) For the inhibition of XO-generated early ROS, cells were pretreated with 200 μM of allopurinol for 1 h and cotreated with 20 μM of 6r for an additional 4 h. The intracellular cellular ROS level was determined using DCFH-DA and was quantified by flow cytometry. The data represent the mean ± SD of 3 independent experiments. Differences were found to be statistically significant at *P < 0.05 (B) After pretreatment with 200 μM of allopurinol for 1 h and cotreatment with 20 μM of 6r for an additional 24 h, the mitochondrial membrane potential was determined using JC-1 and quantified by flow cytometry. The data represent the mean ± SD of 3 independent experiments. Differences were found to be statistically significant at *P < 0.05 (C) Reducing the XO-generated early ROS level by allopurinol attenuated mitochondria-specific ROS generation. After pretreatment with 200 μM of allopurinol for 1 h and cotreatment with 20 μM of 6r for 24 h, the mitochondria-specific ROS level was determined using MitoSOX and quantified by flow cytometry. (D) Allopurinol treatment reduces Bax translocation and cytochrome c release in 6r-treated cells. Cells were pretreated with 200 μM of allopurinol for 1 h, and then cotreated with 20 μM of 6r for 24 h. Mitochondrial and cytosolic fractions were prepared and subjected to western blot analysis for cytochrome c, Bax, ACTIN, and COX-IV.

The XO-dependent early ROS were visible as early as during mitochondrial damage and the mitochondrial apoptotic signaling cascade. This observation implied that XO-generated early ROS in 6r-treated cells can act as a positive effector to cause mitochondrial damage and subsequently trigger mitochondria-dependent apoptotic cell death. To examine the upstream role of early ROS in downstream mitochondrial apoptotic signaling, an XO-specific inhibitor, allopurinol, was used to attenuate early ROS generation in 6r-treated cells. As shown in Fig 5A, the level of 6r-induced early ROS decreased when cells were pretreated with 200 μM of allopurinol. Remarkably, the level of 6r-induced mitochondrial membrane depolarization was effectively reduced when allopurinol was applied to reduce the level of early ROS (Fig 5B). In addition, attenuating early ROS generation with allopurinol also reduced the level of mitochondria-dependent late ROS generation, which was determined using MitoSOX Red (Fig 5C). We further investigated whether reducing the level of early ROS can subsequently suppress the translocation of Bax and the release of cytochrome c. As shown in Fig 5D, cells treated with allopurinol alone did not result in the translocation of the Bax protein into the mitochondria or the release of cytochrome c to the cytoplasm. Instead, 6r treatment increased the levels of the translocation of Bax and the release of cytochrome c. Importantly, the levels of 6r-induced Bax translocation and cytochrome c release were suppressed following allopurinol treatment. Collectively, these experiments showed that 6r-induced early ROS is responsible for subsequent mitochondrial damage and mitochondria-dependent late ROS generation.

**Fig 6:**
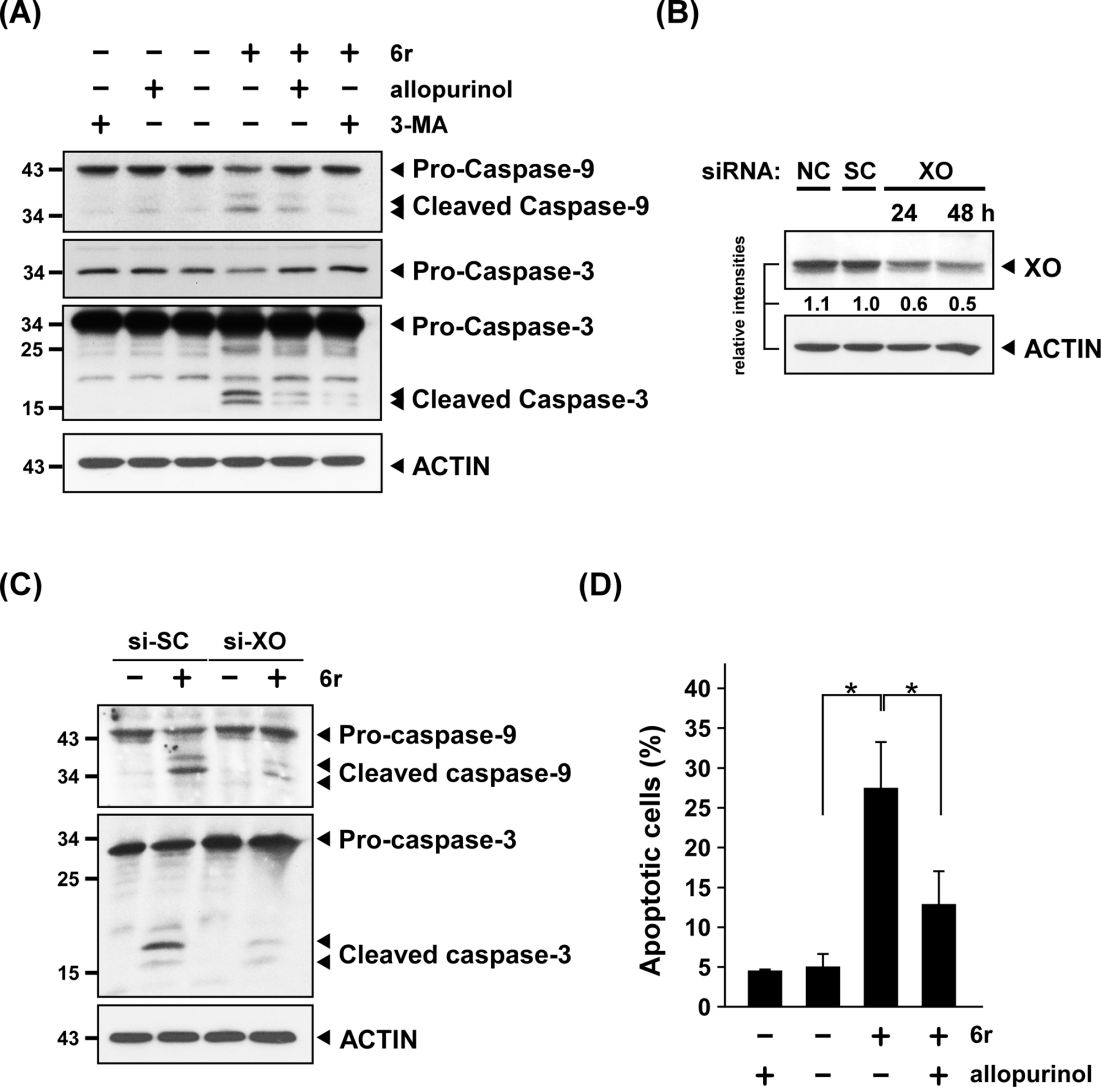
CTSS inhibition-induced early ROS contributes to mitochondria-dependent apoptotic signaling. (A) Allopurinol treatment reduced 6r-induced caspase-9/-3 activation. Cells with and without allopurinol pretreatment were incubated with 20 μM of 6r for 24 h, and subjected to western blot analysis for detecting cleaved caspase-9 and caspase-3. (B) Inhibition of XO expression by siRNA silencing approach. Cells transfected only with RNAiMax was used as a negative control (siNC) and transfected with scramble negative siRNA was used as a scramble control (siSC). (C) Reducing the expression of XO by siRNA decreased 6r-induced caspase-9/-3 activation. After 48 h of siRNA knockdown of XO, cells were treated with 20 μM of 6r for 24 h and the levels of cleaved caspase-9/-3 were assessed by western blotting. (D) Allopurinol treatment reduced the number of 6r-induced apoptotic cells. After pretreatment with allopurinol for 1 h and cotreatment with 6r for 24 h, the numbers of apoptotic cells were determined by Annexin V/PI double staining and quantified by flow cytometry. Differences were found to be statistically significant at *P < 0.05.

The release of cytochrome c initiates the intrinsic apoptotic signaling pathway through caspase-9/-3 activation [41]. To evaluate whether early ROS generation played a crucial role in triggering apoptosis via the intrinsic mitochondrial pathway, we examined the caspase-9/-3 signaling cascade by using western blotting. As shown in Fig 6A, 6r-induced caspase-9 activation was weakened when allopurinol was applied to reduce the upstream early ROS level. In addition, the level of caspase-3 activation in 6r-treating cells consistently decreased. To further confirm if XO is the upstream effector in 6r-induced caspase-9/-3 activation, XO was silenced by specific siRNA and the caspase-9/-3 activation was assessed using western blotting. Suppression of XO was established 24 h after siRNA transfection and the effect was maintained for up to 48 h (Fig 6B). Next, the 6r-induced caspase-9/-3 activation was assessed in both scramble- and XO-silencing cells. In consistent to the results of allopurinol treatment (Fig 6A), 6r-induced caspase-9/-3 activation was attenuated when cells were transfected with XO siRNA (Fig 6C). Because a reduction in the upstream early-ROS level was observed to attenuate the downstream activation of the caspase-9/-3 signaling pathway, early ROS can be considered responsible for triggering apoptosis. Thus, we further determined and quantified the numbers of apoptotic cells in cells treated with 6r in the presence or absence of allopurinol. Our data showed that the increased number of apoptotic cells in 6r-treated cells decreased when cells were co-treated with allopurinol (Fig 6D). Collectively, these experiments indicated that 6r-induced early ROS generation acts as an early executor to activate mitochondria-dependent signaling, which subsequently leads to apoptosis.

## Discussion

To date, “autophagic cell death” has been vaguely defined. No definite markers are available for determining or characterizing autophagic cell death. Most studies have demonstrated that cytotoxic stimuli cause autophagic cell death because autophagic markers are observed in these dying cells. Moreover, the inhibition of autophagy can reduce the final death rate or delay cell death [22–24]. Nevertheless, the term of “autophagic cell death” remains controversial because whether extensive autophagy in these dying cells is a contributor to cell death and/or the initiator for the induction of the death executor is unclear. It is conceivable that constitutive autophagy may trigger cell death because of a massive degradation of organelles that are necessary for cells. However, the positive contribution of autophagy toward cell death has not been identified. In our previous studies, we have observed that the inhibition of CTSS activities can rapidly induce autophagy for early ROS generation [31]. Early ROS was identified to be generated from XO but not from mitochondria, and was found to be harmful for triggering final cell cytotoxicity. In this study, we performed a long-term follow-up analysis and observed that autophagy was quickly induced before 2 h of treatment with 6r and was uninterruptedly up-regulated even after 24 h of CTSS inhibition (Fig 2A). Although the harmful early ROS was quickly visible within 4 h of CTSS inhibition induced by 6r (Fig 3A), no apoptotic morphology or markers were detected. After only 24 h of treatment with 6r, the cells began to exhibit the apoptotic hallmarks including cell shrinkage, decreased nuclear size, increased Annexin V and TUNEL staining, and caspase-3 activation (Fig 1). These observations prompted us to conduct an in-depth investigation into whether XO-generated early ROS can act as a crucial early effector for late mitochondrial damage, subsequently leading to the activation of the mitochondrial apoptotic pathway.

In the present study, we found an accumulation of LC3-II in cells after prolonged 6r treatment. However, one can argue that defective autophagy rather than activated autophagy was induced in 6r-treated OEC-M1 cells. Thus, it is obligatory to determine the autophagic flux in 6r-treated OEC-M1 cells by examining the level of p62/SQSTM, which specially turned over within lysosomes. In addition, autophagic flux was confirmed by using BAF and CQ, which inhibit the late degradation stage of autophagy. Co-treatment with BAF or CQ effectively inhibited p62 degradation and also induced a higher accumulation of LC3-II compared with merely 6r-treated cells. These data clearly confirm that the autophagic flux was activated in 6r-treated OEC-M1 cells.

MitoSOX is a good hydroethidine-based indicator for mitochondrial superoxide measurement by selectively targeting to mitochondria. Once in the mitochondria, MitoSOX is preferentially oxidized by mitochondrial radicals and exhibits red fluorescence. However, it is possible that MitoSOX could be oxidized by other radicals outside of mitochondria in the presence of DNA [42]. To address this concern, it is necessary to examine whether the detected fluorescent MitoSOX signal was localized in mitochondria. As expected, the MitoSOX signal was observed in the cytosol but not in the nucleus, excluding the possible artifacts of nuclear localization of the oxidation products. Additionally, the fluorescent MitoSOX was fully colocalized with mitochondria, as visualized with CytoPainter MitoGreen, confirming that mitochondrial ROS was generated after prolonged 6r treatment.

The mitochondrial signaling pathway that initiates apoptosis involves different environmental stress that damages the mitochondria and induces intracellular signaling cascades [34]. The Bcl-2 family of proteins tightly controls and regulates mitochondrial apoptotic signaling by governing the mitochondrial membrane permeability [43]. Bax, the first identified proapoptotic member of the Bcl-2 protein family, has been reported to translocate from the cytosol to the mitochondria following the exposure of cells to apoptotic stresses [44]. Activated Bax then forms a proteolipid pore in the mitochondrial outer membrane to allow cytochrome c release from mitochondria. This process also facilitates proton leakage and ultimately leads to a mitochondria-dependent oxidative burst. The release of cytochrome c can promote the formation of apoptosomes, which recruit and activate the caspase-9/-3 signaling cascade [41]. In this study, we observed increased mitochondrial levels of Bax protein in 6r-treated cells accompanied by the release of cytochrome c, the decrement of mitochondrial membrane potential, and consequently the activation of caspase-9/-3 signaling pathway. However, whether early ROS generated from autophagy-regulated XO can act as an early effector for triggering apoptosis, or if it is merely an intermediate event that occurs parallel to autophagic/apoptotic cell death remains unclear. Remarkably, reducing XO-generated early ROS by allopurinol not only reduced 6r-induced mitochondrial damage but also attenuated downstream caspase-9/-3 activation. As concerns about the specificity of allopurinol, a complementary biochemical molecular approach was also performed. Consistently, reducing XO expression by siRNA also attenuates 6r-induced caspase-9/-3 activation (Fig 6D). These data indicate that early ROS is not an intermediate event that occurs parallel with 6r-induced autophagic/apoptotic cell death. Instead, autophagy-regulated early ROS is a crucial early effector for late mitochondrial damage and for the activation of the mitochondrial apoptotic pathway.

The number of apoptotic cells induced by 6r treatment decreased by approximately 60% in the presence of an XO inhibitor, allopurinol (Fig 6D). The incomplete prevention of apoptosis by allopurinol may be explained by amplified enzymatic kinetics. Although reducing the early ROS level by using allopurinol can attenuate caspase-3 activation, a small amount of caspase-3 was still activated (Fig 6A). A small amount of activated caspase-3 may be sufficient to induce apoptotic cell death along its enzymatic pathway. Moreover, the harmful characteristics of early ROS may be another critical cause for the failure of allopurinol in the complete prevention of apoptosis. This assumption is derived from our previous work, which demonstrated that early ROS is harmful and can cause DNA damage within 4 h [31]. In addition, XO-generated ROS was recently reported to be sufficient for causing DNA double-strand breaks in cigarette smoke-induced lung injury [45]. In response to DNA damage, cells may trigger the DNA repair system for cell survival. If this fails, the cells can activate caspase-3-independent apoptosis through caspase-2, the only caspase in the nucleus [46, 47]. Thus, the harmful early ROS not only damages the mitochondria for triggering the mitochondria-dependent apoptotic pathway but also damages cellular DNA through the activation of mitochondria-independent apoptosis.

**Fig 7:**
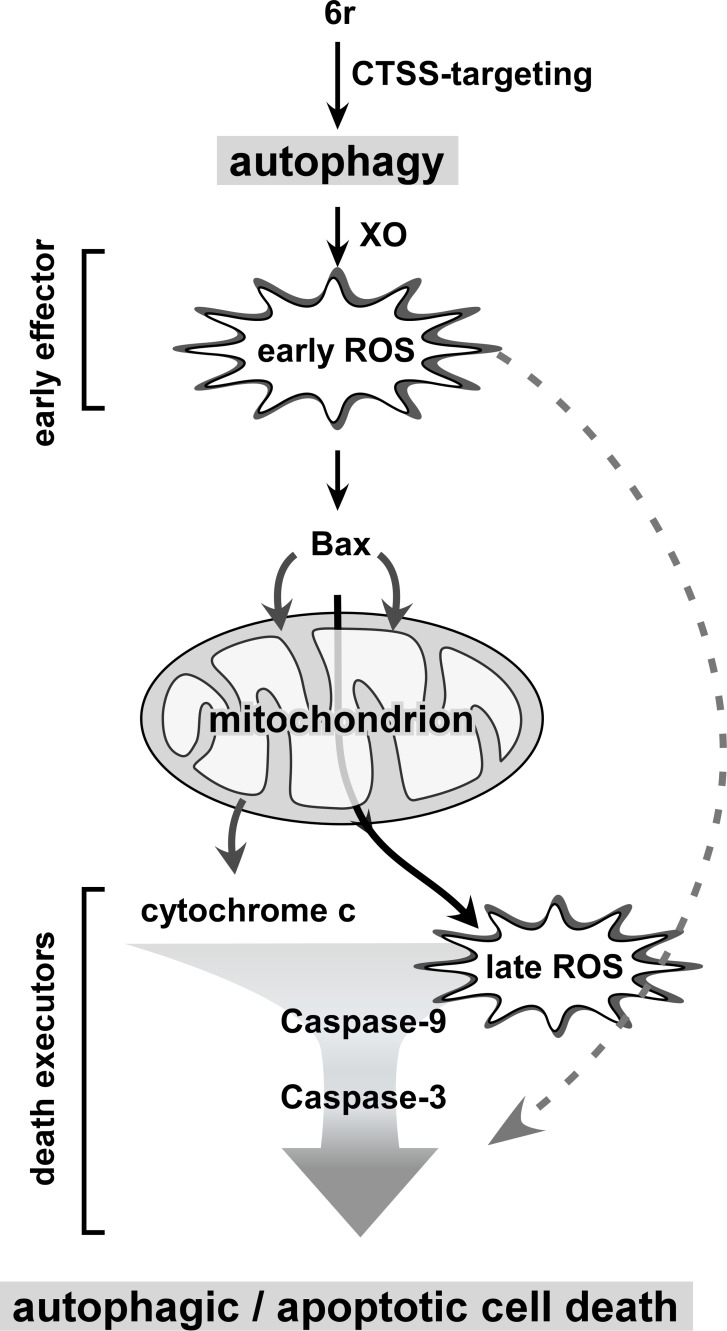
Conclusion model of autophagy-regulated early ROS in the connection between death autophagy and apoptosis. In CTSS-targeting cells, autophagy is quickly activated to regulate XO for the first oxidative burst (early ROS generation). The harmful early ROS can subsequently act as an early effector to for triggering Bax activation and subsequently causing cytochrome c release, and mitochondrial membrane depolarization for the second oxidative burst. Consequently, these factors act as death executors for the triggering of mitochondria-dependent apoptosis. Thus, these dying cells simultaneously display mixed autophagic and apoptotic hallmarks. When upstream autophagy and/or early ROS level are attenuated, mitochondria-dependent apoptotic signaling cascades diminish.

In summary, our findings demonstrated that XO-generated early ROS is a primary event for the connection between autophagic and apoptotic cell death. Following CTSS inhibition by 6r, early ROS is generated from autophagy-regulated XO, and is harmful because it acts as an early effector for triggering Bax activation. Increased Bax translocation to mitochondria was accompanied by the disruption of mitochondrial membrane potential and cytochrome c releasing. Consequently, these factors act as cytotoxic death executors that trigger mitochondria-dependent cell death or intrinsic apoptotic cell death (Fig 7). Thus, inhibiting autophagy (which acts as an upstream event) or reducing the early ROS level (early effector) can attenuate subsequent mitochondrial damage and eventually lower the final death rate (autophagic/apoptotic cell death). Thus, our findings provide innovative connective evidence between death autophagy and apoptosis, showing that mitochondria-dependent apoptosis is triggered by autophagy-regulated early ROS derived from XO in cancer cells treated with CTSS inhibitors.

## References

[1] Palermo C, Joyce JA. Cysteine cathepsin proteases as pharmacological targets in cancer. Trends Pharmacol Sci. 2008;29(1):22–8. Epub 2007/11/27. S0165-6147(07)00274-X [pii] 10.1016/j.tips.2007.10.011 . 18037508

[2] Fernandez PL, Farre X, Nadal A, Fernandez E, Peiro N, Sloane BF, et al Expression of cathepsins B and S in the progression of prostate carcinoma. Int J Cancer. 2001;95(1):51–5. Epub 2001/03/10. 10.1002/1097-0215(20010120)95:1<51::AID-IJC1009>3.0.CO;2-J [pii]. . 11241311

[3] Gocheva V, Zeng W, Ke D, Klimstra D, Reinheckel T, Peters C, et al Distinct roles for cysteine cathepsin genes in multistage tumorigenesis. Genes Dev. 2006;20(5):543–56. Epub 2006/02/17. gad.1407406 [pii] 10.1101/gad.1407406 . 16481467 PMC1410800

[4] Gormley JA, Hegarty SM, O'Grady A, Stevenson MR, Burden RE, Barrett HL, et al The role of Cathepsin S as a marker of prognosis and predictor of chemotherapy benefit in adjuvant CRC: a pilot study. Br J Cancer. 2011;105(10):1487–94. Epub 2011/10/13. bjc2011408 [pii] 10.1038/bjc.2011.408 . 21989182 PMC3242524

[5] Driessen C, Bryant RA, Lennon-Dumenil AM, Villadangos JA, Bryant PW, Shi GP, et al Cathepsin S controls the trafficking and maturation of MHC class II molecules in dendritic cells. J Cell Biol. 1999;147(4):775–90. Epub 1999/11/24. . 10562280 10.1083/jcb.147.4.775 PMC2156161

[6] Shi GP, Villadangos JA, Dranoff G, Small C, Gu L, Haley KJ, et al Cathepsin S required for normal MHC class II peptide loading and germinal center development. Immunity. 1999;10(2):197–206. Epub 1999/03/11. doi: S1074-7613(00)80020-5 [pii]. . 10072072 10.1016/s1074-7613(00)80020-5

[7] Flannery T, Gibson D, Mirakhur M, McQuaid S, Greenan C, Trimble A, et al The clinical significance of cathepsin S expression in human astrocytomas. Am J Pathol. 2003;163(1):175–82. Epub 2003/06/24. S0002-9440(10)63641-3 [pii] 10.1016/S0002-9440(10)63641-3 . 12819022 PMC1868175

[8] Flannery T, McQuaid S, McGoohan C, McConnell RS, McGregor G, Mirakhur M, et al Cathepsin S expression: An independent prognostic factor in glioblastoma tumours—A pilot study. Int J Cancer. 2006;119(4):854–60. Epub 2006/03/22. 10.1002/ijc.21911 . 16550604

[9] Lindahl C, Simonsson M, Bergh A, Thysell E, Antti H, Sund M, et al Increased levels of macrophage-secreted cathepsin S during prostate cancer progression in TRAMP mice and patients. Cancer Genomics Proteomics. 2009;6(3):149–59. Epub 2009/06/03. 6/3/149 [pii]. . 19487544

[10] Paraoan L, Gray D, Hiscott P, Garcia-Finana M, Lane B, Damato B, et al Cathepsin S and its inhibitor cystatin C: imbalance in uveal melanoma. Front Biosci (Landmark Ed). 2009;14:2504–13. Epub 2009/03/11. 3393 [pii]. . 19273215 10.2741/3393

[11] Xu J, Li D, Ke Z, Liu R, Maubach G, Zhuo L. Cathepsin S is aberrantly overexpressed in human hepatocellular carcinoma. Mol Med Rep. 2009;2(5):713–8. Epub 2009/09/01. 10.3892/mmr_00000161. 10.3892/mmr_00000161 21475890

[12] Lee-Dutra A, Wiener DK, Sun S. Cathepsin S inhibitors: 2004–2010. Expert Opin Ther Pat. 2011;21(3):311–37. Epub 2011/02/24. 10.1517/13543776.2011.553800 . 21342054

[13] Chen JC, Uang BJ, Lyu PC, Chang JY, Liu KJ, Kuo CC, et al Design and synthesis of alpha-ketoamides as cathepsin S inhibitors with potential applications against tumor invasion and angiogenesis. J Med Chem. 2010;53(11):4545–9. Epub 2010/05/21. 10.1021/jm100089e . 20481438

[14] Elie BT, Gocheva V, Shree T, Dalrymple SA, Holsinger LJ, Joyce JA. Identification and pre-clinical testing of a reversible cathepsin protease inhibitor reveals anti-tumor efficacy in a pancreatic cancer model. Biochimie. 2010;92(11):1618–24. Epub 2010/05/08. S0300-9084(10)00173-2 [pii] 10.1016/j.biochi.2010.04.023 . 20447439 PMC3814225

[15] Kwok HF, Buick RJ, Kuehn D, Gormley JA, Doherty D, Jaquin TJ, et al Antibody targeting of Cathepsin S induces antibody-dependent cellular cytotoxicity. Mol Cancer. 2011;10:147 Epub 2011/12/16. 1476-4598-10-147 [pii] 10.1186/1476-4598-10-147 . 22168338 PMC3267679

[16] Ward C, Kuehn D, Burden RE, Gormley JA, Jaquin TJ, Gazdoiu M, et al Antibody targeting of cathepsin S inhibits angiogenesis and synergistically enhances anti-VEGF. PLoS One. 2010;5(9). Epub 2010/09/09. 10.1371/journal.pone.0012543 . 20824056 PMC2932732

[17] Burden RE, Gormley JA, Jaquin TJ, Small DM, Quinn DJ, Hegarty SM, et al Antibody-mediated inhibition of cathepsin S blocks colorectal tumor invasion and angiogenesis. Clin Cancer Res. 2009;15(19):6042–51. Epub 2009/10/01. doi: 1078-0432.CCR-09-1262 [pii]10.1158/1078-0432.CCR-09-1262. . 19789302 10.1158/1078-0432.CCR-09-1262

[18] Tooze SA, Yoshimori T. The origin of the autophagosomal membrane. Nat Cell Biol. 2010;12(9):831–5. Epub 2010/09/03. ncb0910-831 [pii] 10.1038/ncb0910-831 . 20811355

[19] Kourtis N, Tavernarakis N. Autophagy and cell death in model organisms. Cell Death Differ. 2009;16(1):21–30. Epub 2008/12/17. cdd2008120 [pii] 10.1038/cdd.2008.120 . 19079286

[20] Lum JJ, DeBerardinis RJ, Thompson CB. Autophagy in metazoans: cell survival in the land of plenty. Nat Rev Mol Cell Biol. 2005;6(6):439–48. Epub 2005/06/02. nrm1660 [pii] 10.1038/nrm1660 . 15928708

[21] Yorimitsu T, Nair U, Yang Z, Klionsky DJ. Endoplasmic reticulum stress triggers autophagy. J Biol Chem. 2006;281(40):30299–304. Epub 2006/08/12. M607007200 [pii] 10.1074/jbc.M607007200 . 16901900 PMC1828866

[22] Kanzawa T, Kondo Y, Ito H, Kondo S, Germano I. Induction of autophagic cell death in malignant glioma cells by arsenic trioxide. Cancer Res. 2003;63(9):2103–8. Epub 2003/05/03. . 12727826

[23] Daido S, Kanzawa T, Yamamoto A, Takeuchi H, Kondo Y, Kondo S. Pivotal role of the cell death factor BNIP3 in ceramide-induced autophagic cell death in malignant glioma cells. Cancer Res. 2004;64(12):4286–93. Epub 2004/06/19. 10.1158/0008-5472.CAN-03-308464/12/4286 [pii]. . 15205343

[24] Elgendy M, Sheridan C, Brumatti G, Martin SJ. Oncogenic Ras-induced expression of Noxa and Beclin-1 promotes autophagic cell death and limits clonogenic survival. Mol Cell. 2011;42(1):23–35. Epub 2011/03/01. S1097-2765(11)00092-X [pii] 10.1016/j.molcel.2011.02.009 . 21353614

[25] Edinger AL, Thompson CB. Death by design: apoptosis, necrosis and autophagy. Curr Opin Cell Biol. 2004;16(6):663–9. Epub 2004/11/09. S0955-0674(04)00147-4 [pii] 10.1016/j.ceb.2004.09.011 . 15530778

[26] Levin S, Bucci TJ, Cohen SM, Fix AS, Hardisty JF, LeGrand EK, et al The nomenclature of cell death: recommendations of an ad hoc Committee of the Society of Toxicologic Pathologists. Toxicol Pathol. 1999;27(4):484–90. Epub 1999/09/15. . 10485836 10.1177/019262339902700419

[27] Codogno P, Meijer AJ. Autophagy and signaling: their role in cell survival and cell death. Cell Death Differ. 2005;12 Suppl 2:1509–18. Epub 2005/10/26. 4401751 [pii] 10.1038/sj.cdd.4401751 . 16247498

[28] Xue L, Fletcher GC, Tolkovsky AM. Autophagy is activated by apoptotic signalling in sympathetic neurons: an alternative mechanism of death execution. Mol Cell Neurosci. 1999;14(3):180–98. Epub 1999/11/30. S1044-7431(99)90780-7 [pii] 10.1006/mcne.1999.0780 . 10576889

[29] Denton D, Nicolson S, Kumar S. Cell death by autophagy: facts and apparent artefacts. Cell Death Differ. 2012;19(1):87–95. Epub 2011/11/05. cdd2011146 [pii] 10.1038/cdd.2011.146 . 22052193 PMC3252836

[30] Chen KL, Chang WS, Cheung CH, Lin CC, Huang CC, Yang YN, et al Targeting cathepsin S induces tumor cell autophagy via the EGFR-ERK signaling pathway. Cancer Lett. 2011;317(1):89–98. Epub 2011/11/22. S0304-3835(11)00715-4 [pii] 10.1016/j.canlet.2011.11.015 . 22101325

[31] Huang CC, Chen KL, Cheung CH, Chang JY. Autophagy induced by cathepsin S inhibition induces early ROS production, oxidative DNA damage, and cell death via xanthine oxidase. Free Radic Biol Med. 2013;65:1473–86. Epub 2013/07/31. S0891-5849(13)00351-1 [pii] 10.1016/j.freeradbiomed.2013.07.020 . 23892358

[32] Meng CL, Yang CY, Shen KL, Wong PY, Lee HK. Inhibition of the synthesis of eicosanoid-like substances in a human oral cancer cell line by interferon-gamma and eicosapentaenoic acid. Arch Oral Biol. 1998;43(12):979–86. Epub 1999/01/07. doi: S0003996998000788 [pii]. . 9877329 10.1016/s0003-9969(98)00078-8

[33] Yang CY, Meng CL. Regulation of PG synthase by EGF and PDGF in human oral, breast, stomach, and fibrosarcoma cancer cell lines. J Dent Res. 1994;73(8):1407–15. Epub 1994/08/01. . 8083436 10.1177/00220345940730080301

[34] Elmore S. Apoptosis: a review of programmed cell death. Toxicol Pathol. 2007;35(4):495–516. Epub 2007/06/15. 779478428 [pii] 10.1080/01926230701320337 . 17562483 PMC2117903

[35] Munafo DB, Colombo MI. A novel assay to study autophagy: regulation of autophagosome vacuole size by amino acid deprivation. J Cell Sci. 2001;114(Pt 20):3619–29. Epub 2001/11/15. . 11707514 10.1242/jcs.114.20.3619

[36] Salvioli S, Ardizzoni A, Franceschi C, Cossarizza A. JC-1, but not DiOC6(3) or rhodamine 123, is a reliable fluorescent probe to assess delta psi changes in intact cells: implications for studies on mitochondrial functionality during apoptosis. FEBS Lett. 1997;411(1):77–82. Epub 1997/07/07. doi: S0014-5793(97)00669-8 [pii]. . 9247146 10.1016/s0014-5793(97)00669-8

[37] Smiley ST, Reers M, Mottola-Hartshorn C, Lin M, Chen A, Smith TW, et al Intracellular heterogeneity in mitochondrial membrane potentials revealed by a J-aggregate-forming lipophilic cation JC-1. Proc Natl Acad Sci U S A. 1991;88(9):3671–5. Epub 1991/05/01. . 2023917 10.1073/pnas.88.9.3671 PMC51514

[38] Heiskanen KM, Bhat MB, Wang HW, Ma J, Nieminen AL. Mitochondrial depolarization accompanies cytochrome c release during apoptosis in PC6 cells. J Biol Chem. 1999;274(9):5654–8. Epub 1999/02/20. . 10026183 10.1074/jbc.274.9.5654

[39] Smaili SS, Hsu YT, Sanders KM, Russell JT, Youle RJ. Bax translocation to mitochondria subsequent to a rapid loss of mitochondrial membrane potential. Cell Death Differ. 2001;8(9):909–20. Epub 2001/08/30. 10.1038/sj.cdd.4400889 . 11526446

[40] Wolter KG, Hsu YT, Smith CL, Nechushtan A, Xi XG, Youle RJ. Movement of Bax from the cytosol to mitochondria during apoptosis. J Cell Biol. 1997;139(5):1281–92. Epub 1998/01/07. . 9382873 10.1083/jcb.139.5.1281 PMC2140220

[41] Kumar S. Caspase function in programmed cell death. Cell Death Differ. 2007;14(1):32–43. Epub 2006/11/04. 4402060 [pii] 10.1038/sj.cdd.4402060 . 17082813

[42] Robinson KM, Janes MS, Pehar M, Monette JS, Ross MF, Hagen TM, et al Selective fluorescent imaging of superoxide in vivo using ethidium-based probes. Proc Natl Acad Sci U S A. 2006;103(41):15038–43. Epub 2006/10/04. 0601945103 [pii] 10.1073/pnas.0601945103 . 17015830 PMC1586181

[43] Cory S, Adams JM. The Bcl2 family: regulators of the cellular life-or-death switch. Nat Rev Cancer. 2002;2(9):647–56. Epub 2002/09/05. 10.1038/nrc883 [pii]. . 12209154

[44] Tait SW, Green DR. Mitochondria and cell death: outer membrane permeabilization and beyond. Nat Rev Mol Cell Biol. 2010;11(9):621–32. Epub 2010/08/05. nrm2952 [pii] 10.1038/nrm2952 . 20683470

[45] Kim BS, Serebreni L, Hamdan O, Wang L, Parniani A, Sussan T, et al Xanthine oxidoreductase is a critical mediator of cigarette smoke-induced endothelial cell DNA damage and apoptosis. Free Radic Biol Med. 2013;60:336–46. Epub 2013/02/06. S0891-5849(13)00033-6 [pii] 10.1016/j.freeradbiomed.2013.01.023 . 23380026

[46] Roos WP, Kaina B. DNA damage-induced cell death: from specific DNA lesions to the DNA damage response and apoptosis. Cancer Lett. 2013;332(2):237–48. Epub 2012/01/21. S0304-3835(12)00032-8 [pii] 10.1016/j.canlet.2012.01.007 . 22261329

[47] Roos WP, Kaina B. DNA damage-induced cell death by apoptosis. Trends Mol Med. 2006;12(9):440–50. Epub 2006/08/11. S1471-4914(06)00168-7 [pii] 10.1016/j.molmed.2006.07.007 . 16899408

